# Dr. Tousley B. Lewis

**Published:** 1903-03

**Authors:** 


Dr. Tousley B. Lewis, of New York, U. of B. '79, died at his home No. 46 West 93d Street, February 8, 1903. The remains were removed to Albion for interment.

